# Age distribution and mortality associated with intussusception in children under two years of age in nine sentinel surveillance hospitals in Zambia, 2007-2018

**DOI:** 10.11604/pamj.supp.2021.39.1.26671

**Published:** 2021-07-28

**Authors:** Evans Mwila Mpabalwani, Bruce Bvulani, Julia Simwaka, Pearson Chitambala, Belem Matapo, Jacqueline Tate, Umesh Parashar, Jason Mathiu Mwenda

**Affiliations:** 1 University of Zambia, School of Medicine, Department of Paediatrics & Child Health, Lusaka, Zambia,; 2 University Teaching Hospitals, Children´s Hospital, Lusaka, Zambia,; 3 University Teaching Hospitals, Adult Hospital, Department of Surgery, Paediatric Surgical Unit, Lusaka, Zambia,; 4 University Teaching Hospitals, Adult Hospital, Virology Laboratory, Lusaka, Zambia,; 5 World Health Organisation, Regional Office for Africa, Zambia & Congo Brazzaville,; 6 Division of Viral Diseases, Centers for Disease Control and Prevention, Atlanta, GA, USA

**Keywords:** Intussusception, surveillance, CFR, Zambia

## Abstract

**Introduction:**

recipients of monovalent rotavirus vaccine have a low risk of developing intussusception (IS) in high- to medium-high-income countries. In sub-Saharan Africa, Zambia included, this risk of IS has not been assessed. Two-dose monovalent rotavirus vaccine, introduced in Zambia in 2012 in the capital of Lusaka, and rolled out countrywide in 2013, is administered at 6 and 10 weeks of age with no catch-up dose. Active IS surveillance monitoring in children < 2 years has been ongoing in Zambia since July 2009 and additional retrospective review was conducted from 2007- June 2009.

**Methods:**

retrospective review (January 2007-June 2009) and prospective (July 2009-December 2018) IS surveillance was conducted at nine hospitals and four large paediatric hospital departments in Zambia, respectively. Demographic and clinical data were collected from medical folder abstraction and supplemented by parental interview during prospective surveillance.

**Results:**

a total of 248 children < 2 years with IS were identified; 57.3% were male. Most cases with IS were infants (85.5%). IS admissions remained stable during the surveillance period with no seasonality pattern although an increase in cases occurred between August and October, hot dry season. The median time from symptom onset to presentation for treatment was 2 days and 63.6% (154/242) of IS diagnoses were made during surgery. The bowel resection rate was 46.6%. A high CFR of 23.3% was observed.

**Conclusion:**

the number of intussusception cases in Zambia was relatively small and remained stable over the 12-year study period. However, a high CFR was observed among cases.

## Introduction

Intussusception (IS), the invagination of a proximal part of the intestine into the distal portion, is the leading cause of intestinal obstruction in young children [1]. To assess IS frequency in Zambia, retrospective review (January 2007-June 2009) and prospective (July 2009-June 2012) surveillance was conducted for IS in children less than 2 years in nine Zambian hospitals [2]. IS naturally occurs among infants and young children in Zambia and the peak age is 5-6 months with high case fatality ratio (CFR) of 33.7%. The high CFR was due to both delayed presentation and delayed diagnosis in hospitals [2].

Zambia introduced monovalent rotavirus vaccine (RVV) in the Expanded Programme on Immunisation (EPI) as a pilot in 2012 in the capital of Lusaka, and was rolled out countrywide in November 2013 with doses given at 6 and 10 weeks of age with no catch-up dose [3]. Through the African Intussusception Surveillance Network, infants with IS from seven low-income countries in sub-Saharan Africa using monovalent RVV, Zambia included, were enrolled and no association of the vaccine and IS was found [4]. However, such an association has been observed in middle- and high-income countries [4]. The benefit/risk profile of monovalent RVV remains strongly positive in low, middle- and high-income countries [4-6] and WHO recommends countries establish and continue with rotavirus sentinel surveillance and IS monitoring as part of post marketing surveillance [7]. In this 12-year surveillance study, we describe the epidemiology of IS in Zambia during the time surrounding rotavirus vaccine introduction.

## Methods

**Study design:** this is a cross-sectional study/retrospective review of IS data. The analysis was conducted as part of the surveillance system for IS conducted as described previously [2].

**Participants and data collection:** the retrospective review of data was conducted by research assistants at nine Zambian Hospitals from January 2007 to June 2009 and July 2009 to June 2011; using theatre log books. Patients below 2 years of age with a diagnosis of IS or intestinal obstruction were identified and had their medical files traced in wards/registry. Demographic and clinical data were abstracted to a standard questionnaire. Active surveillance was continued in a subset of 4 large pediatric hospitals from July 2011 to December 2018 using a standard questionnaire. These four large hospitals included the University Teaching Hospitals (UTHs) Lusaka Children’s, Arthur Davison Children’s Hospital in Ndola, Kitwe Central Hospital, and Livingstone Central Hospital [2]. Children < 2 years of age meeting the Brighton Collaboration Level 1 criteria [8] for diagnostic certainty were enrolled. For all patients, information demographics and clinical characteristics were obtained from medical folder review, for prospectively enrolled participants, through parental interviews. The data is shared with the Ministry of Health, Zambia, and the WHO coordinated African Intussusception Surveillance Network.

**Data analysis:** simple descriptive statistics were used to summarize demographic and clinical information using Epi Info Version 3.5.4.

**Ethical aspects:** the protocol was reviewed by the Zambia MOH and determined to be a public health evaluation. Measures were taken to ensure the protection of participants´ privacy and confidentiality.

## Results

**Figure 1:**
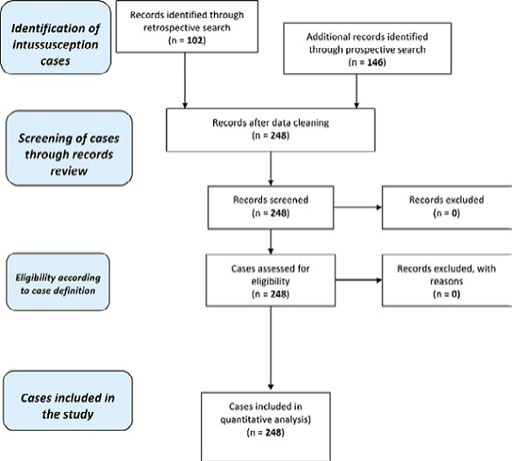
intussusception cases from nine sentinel surveillance Zambian Hospitals, 2007-2018

**Figure 2:**
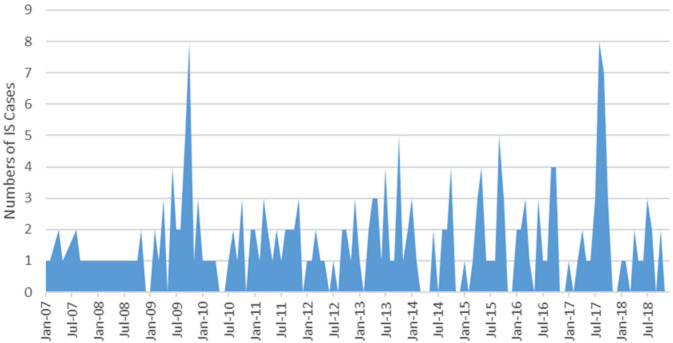
monthly distribution of children < 2 years admitted with intussusception in the surveillance sites in Zambia, 2007-2018

During the 12-year period from January 2007 to December 2018, 248 children < 2 years of age with IS were enrolled with retrospective review and prospective surveillance accounting for 101 and 147 respectively (Table 1). The majority, 57.3% were male (Table 1). Figure 1 shows the flow chart of enrolling children in the study. The majority, 64.9% (161/248) of patients were recruited at UTHs Lusaka Children´s Hospital (Table 1). Of the children < 2 years, most with IS (85.5%) were infants < 1 year of age (Table 1). IS in infants was common in those aged 3-8 months old with a peak age of 6-8 months. The time between onset of symptoms and hospital admission was a median of 2 days. Most of the patients (51.3%) were transferred from another facility. The median length of stay in hospital was 8 days (interquartile range: 4-10 days) (Table 2). The length of stay in hospital did not vary by no more than 8 days and was positively skewed. In the majority (63.6%, 154/242) of children, the diagnosis of IS was made during surgery and the bowel resection rate was 46.6% (Table 2). Alternatively, diagnosis of IS was made on plain abdominal X-ray (23.1%), ultrasound (7.9%), and by using clinical symptoms and signs (5.4%) (Table 2). A high CFR of 23.3% was observed. There was no definite seasonal pattern observed although an increase in cases occurred between August and October, hot dry season (Figure 2).

**Table 1:** surveillance sites and sociodemographic characteristics of children < 2 years with intussusception in Zambia, 2007-2018

Parameter		Medical record search 2007-June 2009 n/N (%)	Prospective cases July 2009-2018 n/N (%)	N= 248 n/N (%)
Surveillance sites				
Arthur Davison Children´s Teaching Hospital, Ndola		11 (10.9)	24 (16.3)	35/248(14.1)
Kitwe Teaching Hospital		6 (5.9)	14 (9.5)	20/248(8.1)
Livingstone Teaching Hospital		8 (7.9)	10 (6.8)	18/248(7.3)
University Teaching Hospital, Lusaka		67 (66.3)	94 (63.9)	161/248(64.9)
Others		9 (8.9)	5 (3.4)	14/248(5.6)
**Total**		101	147	
**Median age in months (range)**		6 months [1-36]		
**Age distribution**	**Infants**	All		
0-2 months	9/212(4)	9/248(3.6)		
3-5 months	84/212(39.6)	84/248(33.9)		
6-8 months	92/212(43.4)	92/248(36.1)		
9-11 months	27/212(12.7)	27/248(10.9)		
Total infants	-	212/248(85.5)		
12-17 months		18/248(7.3)		
18-23 months		6/248(2.4)		
≥24 months		7/248(2.8)		
Missing data		5/248(2.0)		
**Sex, N (%) Male**		142 (57.3%)		
N (%) of children transferred from another facility*		59 (51.3%)		
Median number of days (range) between symptom onset and admission to surveillance facility*		2 days		

*2013-18 data

**Table 2:** diagnosis, treatment and outcome of children < 2 years with intussusception in Zambia, 2007-2018

Diagnosis of intussusception**		n / N(%)
Clinical symptoms		13/242(5.4)
Enema		0
Radiographic X-ray		56/242(23.1)
Ultrasound		19/242(7.9)
Surgery		154/242(63.6)
Missing data		6
Treatment of Intussusception		
Enema		1/245 (0.0)
Surgery		232/245(94.7)
Spontaneous resolution		12/245(0.05)
Missing data		3
Among children with surgery, n (%) that required resection		108/232(46.6)
Median length of hospital-stay in days - interquartile range (IQR) (n=248)		8 [4-10]
Median length of hospital-stay in those with resection [IQR] (n=108)		9 [4-10]
Outcome	Discharged home	176/232(75.9)
	Transferred	0
	Died (CFR)	54/230(23.3)
	Missing data	18

**Diagnostic categories not mutually exclusive; multiple pieces of information could contribute to diagnosis

## Discussion

Of the 248 cases of IS in children < 2 years of age who were enrolled from the initial nine and later four hospitals in Zambia over the 12-year surveillance period, the majority, more than two thirds were from UTHs´ Lusaka Children´s Hospital the largest tertiary facility in the country [2]. There was male preponderance and predominantly infants, 86.5%, this is consistent with other studies [2, 9-11]. The peak age for IS was 6-8 months, and this is outside the age of 6- and 10-week administration of monovalent rotavirus vaccine in Zambia. Non-surgical diagnostic methods for IS were not readily available, as the majority of IS patients were diagnosed during surgery. Plain abdominal X-ray and ultrasound were rarely done as the patients were probably taken for operation due to non-availability of personnel to do ultrasound. Definite clinical diagnosis using clinical symptoms/signs was again rare as most the patients had a diagnosis of acute abdomen needing surgery. Therefore, the main course of treatment for IS in our surveillance system was surgery (94.7%) similar to other settings in the sub-Saharan Africa [9, 10, 12, 13]. This finding contrasts sharply with lower rates of surgical intervention in Europe, and North America where barium/air enema is the main mode of treatment [14]. However, a recent study in Ibadan, Nigeria, has demonstrated that the increased utilisation of ultrasound-guided hydrostatic procedures has reduced surgical intervention in uncomplicated cases [12].

The CFR finding of 23.3% in the current study is high, although lower than what was observed in the previous study (33.7%) [2]. The high CFR can be attributed to high bowel resection rate of 46.6% which is mainly due to nonviable intestine as a result of delayed presentation to the point of care [2, 12]. The high rate of intestinal resection could have been due to transport problems as most patients had to be transferred from another facility and this may have contributed to delayed presentation and treatment [2, 12]. About half of the patients remained admitted in hospital for more than 5 days following surgery and this is most likely due to sepsis following delayed treatment [2]. The marginal decrease in CFR is possibly due to increased awareness of the condition by health workers at various levels of care and training provided during active surveillance although this CFR is still higher than countries in the sub-region [9-11] and other regions globally [14].

This downward trend in mortality is expected to decrease further with increased sensitisation of health workers. However, some children with IS do not make it to the point of care [2, 9] and therefore remain undocumented. In the face of increasing utilisation of RVVs by more than 34 countries in sub-Saharan Africa as at April 2020 [15], probably the Integrated Management of Childhood Illnesses (IMCI) which emphasises a diagnosis of bloody diarrhoea in children as dysentery could include IS as a cause of bloody diarrhoea. The IMCI programme could be an entry point for sensitising health workers in the rural areas where these guidelines are strictly followed [9]. This will lead to early diagnosis and early transfer of patients to hospitals where surgery can be done. In fact, the younger the infant is, a diagnosis of dysentery is highly unlikely unless there is an index case in the family. However, it was observed that there was better outcome of children with IS who presented early to the point of care after the onset of symptoms. This could be attributed to increased awareness of the condition in the latter period of the surveillance.

### Limitations

The limitations to this observational evaluation of IS is the small birth cohort in Zambia of under 600,000 and the limited number of sentinel sites. In the initial part of the surveillance data were collected retrospectively and in the latter period data was collected prospectively. This could account for the differences in the completeness of data. As some hospitals rarely treated IS cases, the number of surveillance sites was decreased from 9 hospitals to the 4 hospitals with the largest enrollments in 2011. These 4 hospitals enrolled almost 95% of the cases included in the analysis. Notwithstanding the rarity of IS, surveillance for IS should continue and be integrated in the national EPI Adverse Events Following Immunisation (AEFI) reporting system.

Disclaimer: the findings and conclusions of this report are those of the authors and do not necessarily represent the official position of the US Centers for Disease Control and Prevention and the World Health Organisation.

## Conclusion

The CFR of IS has significantly declined from previously reported estimates although it remains at a higher rate compared to the sub-regional and global figures. Surgical intervention remains the main stay of treatment but non-operative intervention with the use of ultrasound guided hydrostatic procedures can be considered in uncomplicated IS cases. IS occurs at a lower rate in young infants ≤ 4 months of age and there is need to consider IS as cause of bloody diarrhoea in young infants in the IMCI guidelines. IS monitoring should be integrated in the existing routine AEFI reporting system.

### What is known about this topic

- Intussusception in infants is a rare condition and mortality is high in sub-Saharan Africa;
- Intussusception is diagnosed as dysentery (bloody diarrhoea) in most sub-Saharan countries as per IMCI guidelines.

### What this study adds

- Mortality due to intussusception in infants remains very high in Zambia compared to regional and global figures;
- Intussusception in infants in Zambia occurred early, outside the immunisation age group;
- Apart from dysentery, intussusception should be considered as a cause of bloody diarrhoea in infants in the IMCI guidelines.
